# Silencing of crustacean hyperglycemic hormone gene expression reveals the characteristic energy and metabolic changes in the gills and epidermis of crayfish *Procambarus clarkii*

**DOI:** 10.3389/fphys.2023.1349106

**Published:** 2024-01-10

**Authors:** Wen-Feng Li, Shan Zhang, Kuo-Hsun Chiu, Xiao-Yun Deng, Yi Yi

**Affiliations:** ^1^ Yazhou Bay Innovation Institute, Hainan Tropical Ocean University, Sanya, China; ^2^ Key Laboratory of Utilization and Conservation for Tropical Marine Bioresources, Ministry of Education, Hainan Tropical Ocean University, Sanya, China; ^3^ Hainan Key Laboratory for Conservation and Utilization of Tropical Marine Fishery Resources, Hainan Tropical Ocean University, Sanya, China; ^4^ College of Fisheries and Life Science, Hainan Tropical Ocean University, Sanya, China; ^5^ Department of Biology, National Changhua University of Education, Changhua, Taiwan; ^6^ Department of Aquaculture, National Kaohsiung University of Science and Technology, Kaohsiung, Taiwan

**Keywords:** *Procambarus clarkii*, crustacean hyperglycemic hormone, metabolomics, double-stranded RNA, gills, epidermis

## Abstract

The crustacean hyperglycemic hormone (CHH) is a multifaceted neuropeptide instrumental in regulating carbohydrate and lipid metabolism, reproduction, osmoregulation, molting, and metamorphosis. Despite its significance, there is a dearth of research on its metabolic impact on the gills and epidermis—key organs in osmoregulation and molting processes. This study employed CHH dsRNA injections to silence CHH gene expression in *Procambarus clarkii,* followed by a metabolomic analysis of the gills and epidermis using nuclear magnetic resonance spectroscopy. Metabolic profiling through principal component analysis revealed the most pronounced changes at 24 h post-injection (hpi) in the epidermis and at 48 hpi in the gills. At 24 hpi, the epidermis exhibited significant modulation in 25 enrichment sets and 20 KEGG pathways, while at 48 hpi, 5 metabolite sets and 6 KEGG pathways were prominently regulated. Notably, pathways associated with amino acid metabolism, carbohydrate metabolism, and cofactor and vitamin metabolism were affected. A marked decrease in glucose and other carbohydrates suggested a compromised carbohydrate supply, whereas increased levels of citrate cycle intermediates implied a potential boost in energy provision. The silencing of CHH gene expression hampered the carbohydrate supply, which was possibly the main energy derived substrates. Conversely, the gills displayed significant alterations in 15 metabolite sets and 16 KEGG pathways at 48 hpi, with no significant changes at 24 hpi. These changes encompassed amino acid, carbohydrate, and lipid metabolism pathways. The decline in TCA cycle intermediates pointed to a potential downregulation of the cycle, whereas a decrease in ketone bodies indicated a shift towards lipid metabolism for energy production. Additionally, increased levels of nicotinate, nicotinamide, and quinolinate were observed in both organs. Overall, CHH’s impact on the epidermis was prominent at 24 hpi and diminished thereafter, whereas its influence on metabolism in gills was delayed but intensified at 48 hpi. This differential CHH effect between gills and epidermis in *P. clarkii* provides new insights into the organ-specific regulatory mechanisms of CHH on energy metabolism and osmoregulation, warranting further comparative studies to elucidate the distinct roles of CHH in these organs.

## 1 Introduction

The X-organ/sinus gland (XO/SG) complex, situated within the eyestalks of crustaceans, is responsible for the synthesis and secretion of a series of neuropeptides, also referred to as neurohormones. These compounds play a critical role in regulating a host of physiological functions, including metabolism, molting (ecdysis), reproduction, ionic/osmotic balance, and pigmentation changes (Li et al., 2019). Consequently, the eyestalks, specifically the XO/SG complex, have been acknowledged as one of the pivotal endocrine organs in crustacean physiology (Chen et al., 2020). Over recent decades, extensive research has led to the identification of a variety of functionally diverse neuropeptides within the XO/SG complex of decapods. These include the crustacean hyperglycemic hormone (CHH), molt-inhibiting hormone (MIH), gonad/vitellogenesis-inhibiting hormone (GIH/VIH), and mandibular organ-inhibiting hormone (MOIH). Collectively, these peptide hormones comprise the CHH family (Lee et al., 2014; Chen et al., 2020).

The crustacean hyperglycemic hormone (CHH) is a neuropeptide hormone first identified in the X-organ/sinus gland (XO/SG) complex. Initially dubbed a “diabetogenic factor” in the eyestalks of decapods in 1944 (Abramowitz et al., 1944), CHH has since become the focus of considerable research efforts (Chen et al., 2020). The first complete amino acid sequence of CHH was elucidated from the shore crab *Carcinus maenas*. To date, over eighty CHH or CHH-like peptides have been identified and characterized (Kegel et al., 1989; Nagai et al., 2011; Aquiloni et al., 2012; Manfrin et al., 2013; Liu et al., 2019). These CHH peptides are characteristically composed of 72–73 amino acids, featuring six conserved internal cysteine residues that typically form three characteristic disulfide bridges. Additionally, their amino and carboxyl termini are structurally blocked (Christie et al., 1987).

Currently, CHH is recognized as a pleiotropic hormone, essential for diverse physiological processes such as metabolism, molting, reproduction, ionic/osmotic regulation, and immunity (Chen et al., 2020). A substantial body of research indicates CHH’s primary role in glycogen metabolism—specifically, it enhances glycogen synthase activity while reducing glycogen phosphorylase activity, thus promoting glycogenolysis, leading to hyperglycemia and subsequently increasing glycolytic flux (Sedlmeier, 1987; Chen et al., 2020). Additionally, both *in vivo* and *in vitro* studies have demonstrated CHH’s role in stimulating lipid metabolism (Santos et al., 1997). Eyestalk ablation markedly reduces levels of phospholipids, triglycerides, and free fatty acids in the hemolymph. Conversely, *in vivo* administration of recombinant CHH can restore these lipid levels (Montiel-Arzate et al., 2020). Furthermore, employing RNA interference (RNA*i*) techniques and metabolomic profiling has unveiled CHH’s stimulation of lipolysis in the hepatopancreas, its promotion of carbohydrate utilization through glycolysis and the tricarboxylic acid (TCA) cycle, as well as the enhancement of the pentose phosphate pathway (PPP) flux and amino acid biosynthesis in muscle tissue (Li et al., 2017; Li et al., 2019; Chen et al., 2020). Hence, CHH is pivotal and multifaceted in regulating energy metabolism.

Additionally, the role of CHH in ionic/osmotic homeostasis regulation has emerged as a subject of significant interest (Serrano et al., 2003; Chung and Webster, 2006; Chen et al., 2020). Eyestalk ablation has been observed to precipitate a marked decline in hemolymph osmolality, which results in increased water content and reduced Cl^−^ and Na^+^ concentrations in the hemolymph of species such as *Homarus americanus* (Charmantier-Daures et al., 1994), *Procambarus clarkii* (Kamemoto, 1991), and *Uca pugilator* (Davis and Hagadorn, 1982). In *Pachygrapsus marmoratus*, gill perfusion with CHH consistently and significantly heightened the transepithelial potential difference and augmented Na^+^ influx by approximately 50%, demonstrating a rapid and reversible effect, suggestive of CHH’s involvement in branchial ionic transport regulation (Spanings-Pierrot et al., 2000). Furthermore, in *Astacus leptodactylus*, injection with D-Ph3-CHH significantly elevated hemolymph osmolality and Na^+^ levels within 24 h post-injection, underscoring CHH’s role in osmoregulation in freshwater crustaceans (Serrano et al., 2003).

The mechanisms by which CHH influences osmoregulation remain elusive, and potential targets are believed to be varied, including the permeability of the body surface, excretory organs, and gills (Charmantier-Daures et al., 1994). The specific functions implicated are also not well-defined but are thought to encompass the drinking rate, diuresis, salt uptake, and the activity of Na^+^-K^+^-ATPase (Kamemoto, 1991). One theory posits that CHH may modulate Na^+^-K^+^-ATPase activity via cGMP as a secondary messenger, supported by observations of a 50% increase in Na^+^-K^+^-ATPase activity following CHH perfusion (Spanings-Pierrot et al., 2000).

Another hypothesis contends that CHH enhances the availability of metabolizable energy for ion-exchange pumps via increased glycogenolysis, a theory supported by empirical data: CHH binding sites have been identified on the gills of *Carcinus maenas*, and *in vitro* CHH application on gill tissue markedly elevated both cGMP and glucose levels (Spanings-Pierrot et al., 2000; Chung and Webster, 2006). Nevertheless, the intricacies of CHH’s regulatory effects on ionic/osmotic balance may be more complex. For instance, CHH variants from the Christmas Island blue crab *Discoplax celeste* have been shown to stimulate Na^+^ transport across gill epithelia without influencing the activity of gill Na^+^/K^+^-ATPase or V-ATPase (Turner et al., 2013). Ultimately, the specific mechanisms by which CHH regulates ionic/osmotic homeostasis warrant further investigation.

The aim of this study is to definitively ascertain the role of Crustacean Hyperglycemic Hormone (CHH) in inducing physiological changes within the gills and epidermis of the crayfish *Procambarus clarkii* through metabolomic analysis. Given that the epidermis and gills are external organs in direct contact with the environment, they serve as valuable indicators for comparative stress response studies in both aquaculture and natural settings (Arellano et al., 2004). These organs are multifunctional, playing critical roles in disease resistance, respiration, osmoregulation, and defense against parasites and pathogens (Fletcher, 1978; Shephard, 1994; Arellano et al., 2004). While extensive research has examined the structure and function of fish skin (epidermis) and gills—including mucous, sensory, chloride, and club cells in the epidermis, and water fluxes, gas exchange, acid-base balance, and ionic regulation in gills (Sarasquete et al., 1998; Sarasquete et al., 2001; Arellano et al., 1999; Fernandes and Perna-Martins, 2001; Arellano et al., 2004)—studies on crustacean epidermis and gills, particularly those looking at metabolism and osmoregulation modulated by neurohormones like CHH, remain scarce (Chung and Webster, 2006). Leveraging a previously developed double-stranded RNA (dsRNA)-based gene silencing technique, we were able to substantially reduce CHH levels in the sinus gland and hemolymph (Li et al., 2017). Metabolites in gills and epidermis from control saline-injected and CHH dsRNA-injected specimens were then identified and quantified using nuclear magnetic resonance spectroscopy. The data thus obtained will elucidate the tissue-specific regulatory effects of CHH on both the epidermis and gills.

## 2 Materials and methods

### 2.1 Experimental animals

The crayfish *Procambarus clarkii*, weighing 5.50 ± 0.50 g, utilized in this research were obtained from local fishermen in Xihu River, Miaoli County, Taiwan (24.25° N, 120.45° E). In the laboratory, the specimens were maintained in aquaria (100 cm × 80 cm × 60 cm) with filtered, oxygenated freshwater at a temperature of 24°C ± 1°C, under a 12-h light/12-h dark cycle (Zou et al., 2003). The animal experiments conducted in this study have received full approval from the review committee at National Changhua University of Education (Permit number: NCUE-103320001) and complied with the “Guidelines for Management and Use of Experimental Animals” as recommended by the Council of Agriculture, Taiwan. All crayfish used were confirmed to be in the intermolt stage of their life cycle (Gorgels-Kallen and Voorter, 1985).

### 2.2 Production of Pc-CHH double-strained RNA (dsRNA)

The Pc-CHH dsRNA was synthesized following the methodology outlined by Li et al. (2017). CHH DNA templates for both sense and antisense strands, each incorporating T7 promoter sequences, were amplified using PCR with specific primers as previously detailed (Li et al., 2017). The amplification involved a 25-µL PCR mixture containing 5 µL of 5X Colorless GoTaq Flexi Buffer (Promega), 2.5 µL of 25mM MgCl_2_, 0.5 µL of 10 mM dNTPs, 0.5 µL of 10 µM forward primer, 0.5 µL of 10 µM reverse primer, 0.13 µL of GoTaq DNA polymerase (5U/μL), and 1 µL of eyestalk cDNA. This cDNA was synthesized as described in Li et al. (2017) and stored at −20°C for subsequent use. Post-amplification, the PCR products were purified using a commercial kit (PCR AdvancedTM, VIOGENE) and verified through sequencing. The resultant single-stranded RNAs were then transcribed *in vitro* using the T7 RiboMAXTM Express RNAi System (Promega), following the manufacturer’s protocol. These strands were mixed, denatured at 70°C for 10 min, and annealed by gradually cooling to 25°C at a rate of −1.5°C per hour. The double-stranded RNA was purified, re-suspended in nuclease-free water, and stored at −80°C until needed.

### 2.3 RNA *interference* experiment

A functional knockdown study using Pc-CHH double-stranded RNA (dsRNA) interference was designed to elucidate the role of Pc-CHH in the physiology of gills and epidermis. The experiments were consistently carried out in the morning hours between 9 and 11 a.m. The specimens were divided into two cohorts: the saline-injected (SAI) group and the Pc-CHH dsRNA-injected (DSI) group, with 15 specimens in each. For the injections, the SAI group received 50 µL of HEPES-buffered van Harreveld’s crayfish saline per specimen, pH 7.4, as described by Zou et al. (2003), while the DSI group was administered with 50 µL of Pc-CHH dsRNA solution per specimen, at a concentration of 30 µg of dsRNA per gram of body weight, diluted in crayfish saline. Sampling of gills and epidermis from both the SAI and DSI groups was performed at 24- and 48-h post-injection (hpi), respectively. The tissues collected from three animals were pooled to constitute a single sample, and this process was repeated to obtain five parallel samples. These were then subjected to a heat shock at 100°C for 5 min, followed by rapid freezing in liquid nitrogen, pending nuclear magnetic resonance (NMR) spectroscopy analysis.

### 2.4 ^1^H nuclear magnetic resonance (NMR) analysis

Metabolites from the gills and epidermis were isolated using a modified version of the extraction protocol outlined by Teng et al. (2013). Initially, tissues obtained from both the SAI and DSI groups were rapidly frozen with liquid nitrogen and then pulverized in a ceramic mortar. The resulting powders were homogenized with 2,500 μL/g of 50% methanol, chilled to maintain a temperature of 4°C for 15 min, using a Bullet Blender^®^ (Next Advance). Approximately 250 μL of this homogenate was transferred into a new vial to which an equal volume of 50% methanol and 0.5 mL of chloroform were added. This mixture was then homogenized at 4°C for an additional 20 min and centrifuged at 12,000 g, also at 4°C, for 15 min. The aqueous layer was carefully collected and the extraction solvents were completely evaporated using a vacuum concentrator (Savant SpeedVac, Thermo Scientific). The dry samples were re-dissolved in D_2_O (Merck) containing 1 mM of the sodium salt of 3-(trimethylsilyl) propionate-2,2,3,3-d4 (TSP) in preparation for NMR spectroscopy.

NMR spectra were recorded using a Varian Inova 500 MHz NMR spectrometer (Varian, Inc.) at a controlled temperature of 25°C. The technique of pre-saturation (PRESAT sequence) was employed to suppress the signal from residual water. For each sample, the spectral data was accumulated over 128 scans, spanning a spectral width of 8 kHz, and converted into 32,000 data points. The collected free induction decays (FIDs) were processed with Fourier transformation applying an exponential line-broadening factor of 0.3 Hz to enhance signal clarity, as per Su et al. (2014).

### 2.5 Metabolomic data preprocessing and identification

Metabolomic data were preprocessed and identified using established methods (Li et al., 2017and2019). The NMR spectra underwent phasing, baseline correction, and referencing to TSP (0.0 ppm) before quantification and qualification. These processes were executed utilizing the Chenomx NMR Suite 4.6, which includes the Chenomx 500-MHz library (pH range 6–8) (Chenomx, Inc., Edmonton, Alberta, Canada). It should be noted that the spectral range between 5.0 and 4.7 ppm, which typically exhibits residual water resonance, was deliberately omitted from analysis.

### 2.6 Statistical analysis

Metabolomic profiles were analyzed using MetaboAnalyst 5.0, an online software tool (http://www.metaboanalyst.ca, Chong et al., 2019). Prior to statistical evaluation, the metabolomic data underwent normalization (by median), Log10 transformation, and auto-scaling. The identification of metabolites with statistically significant differences between the DSI and SAI groups was based on the Student’s t-test, considering *p*-values less than 0.05 as indicative of significant variation. These identified metabolites were further subjected to multivariate analysis, including principal component analysis (PCA) and orthogonal projections to latent structures discriminant analysis (oPLS-DA), through MetaboAnalyst 5.0. The robustness of the oPLS-DA model was assessed by a permutation test, conducted 1,000 times.

### 2.7 Metabolite pathway, enrichment, and biomarker analysis

Metabolite pathway analysis (MetPA), metabolite set enrichment analysis (MSEA), and biomarker analysis were carried out using MetaboAnalyst 5.0 (Chong et al., 2019). MetPA and MSEA aimed to pinpoint significantly altered pathways utilizing a metabolite set library associated with pathways from the Kyoto Encyclopedia of Genes and Genomes (KEGG). The methodology for MetPA involved the global test for enrichment, relative-betweenness centrality for topology analysis, and the pathway library corresponding to the fruit fly *Drosophila melanogaster*. Concurrently, MSEA was performed, categorizing metabolites based on the main chemical structures class, which includes 464 principal chemical class metabolite sets.

Biomarker discovery was approached through multivariate exploratory receiver operating characteristic (ROC) analysis. The analysis calculated both ROC curves and the areas under these curves (AUC) to evaluate the discriminative power of individual metabolites. This multivariate exploratory ROC analysis utilized a multivariate algorithm, which employed Monte-Carlo cross-validation (MCCV) for biomarker identification, classification by Partial Least Squares-Discriminant Analysis (PLS-DA), and ranking through the “PLS-DA built-in” feature.

## 3 Results

### 3.1 Metabolomic profiles of gills and epidermis

**FIGURE 1:**
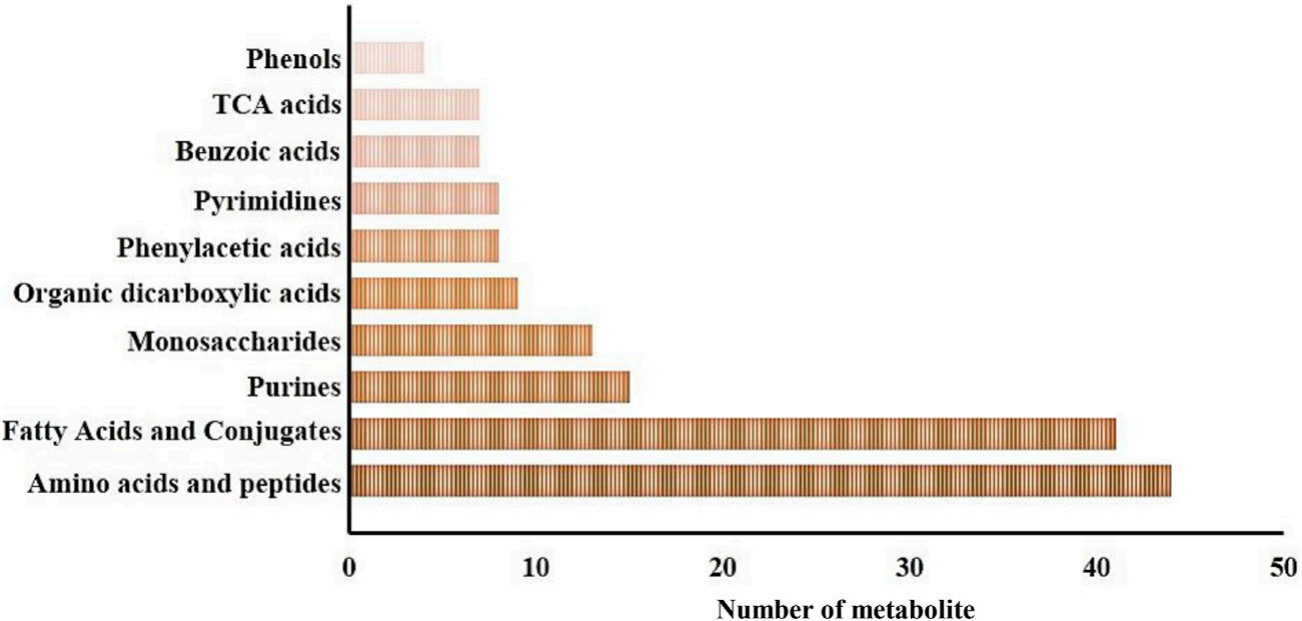
Major metabolite classification for the whole metabolomic profiles of the epidermis and gills of *P. clarkii*.

In this study, a total of 269 metabolites were identified within gills and epidermis samples from the SAI and DSI groups. Metabolite Set Enrichment Analysis (MSEA) with main chemical class metabolite sets revealed 51 metabolite classifications. The top ten categories included amino acids and peptides (44 metabolites), fatty acids and conjugates (41 metabolites), purines (15 metabolites), monosaccharides (13 metabolites), organic dicarboxylic acids (9 metabolites), phenylacetic acids (8 metabolites), pyrimidines (8 metabolites), benzoic acids (7 metabolites), tricarboxylic acid (TCA) cycle acids (7 metabolites), and phenols (4 metabolites), as depicted in Figure 1.

**FIGURE 2:**
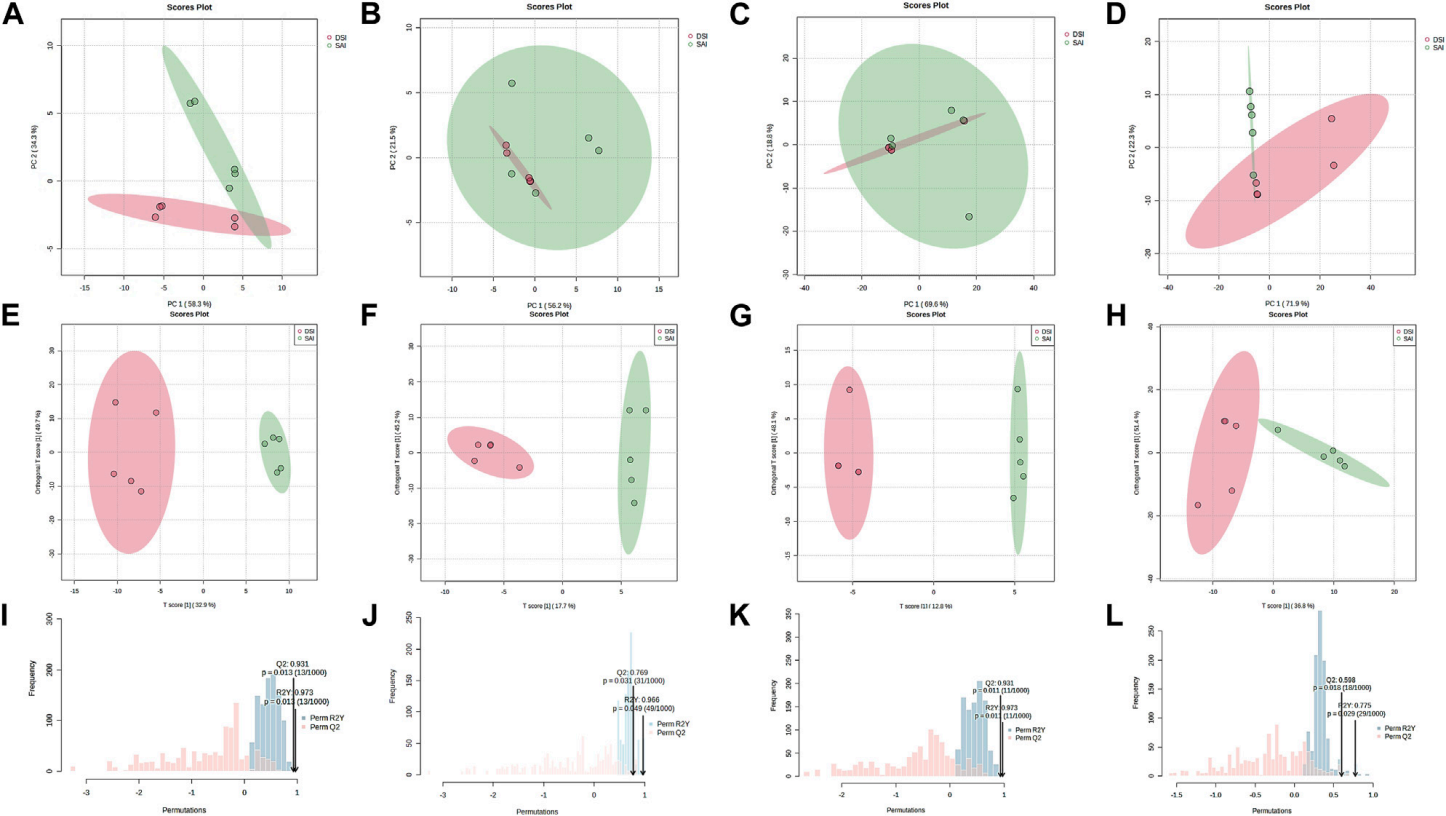
PCA score plot **(A–D)**, oPLS-DA score plot **(E–H)**, and the permutation test for the oPLS-DA **(I–L)** of the epidermis **(A,B,E,F,I,J)** and gills **(C,D,G,H,K,L)** of *P. clarkii* at 24 hpi **(A,C,E,G,I,K)** and 48 hpi **(B,D,F,H,J,L)**.

Principal Component Analysis (PCA) of epidermal metabolites at 24 h post-injection (hpi) demonstrated distinct separation between the SAI and DSI groups (Figure 2A; PC1 and PC2 combined accounted for 92.60% of total variance). However, at 48 hpi, this distinction was less pronounced (Figure 2B; PC1 and PC2 combined accounted for 77.70% of total variance). Conversely, the PCA of gill metabolites did not exhibit clear discrimination at 24 hpi (Figure 2C; PC1 and PC2 combined accounted for 88.40% of total variance), but showed greater differentiation at 48 hpi (Figure 2D; PC1 and PC2 combined accounted for 94.20% of total variance). Orthogonal Partial Least Squares Discriminant Analysis (oPLS-DA) score plots derived from gills and epidermal metabolite profiles indicated robust separation at both 24 and 48 hpi (Figures 2E–H). Permutation testing confirmed the oPLS-DA models were sufficiently descriptive and predictive for the metabolite profiles (Figures 2I–L).

### 3.2 Significantly changed metabolites

**FIGURE 3:**
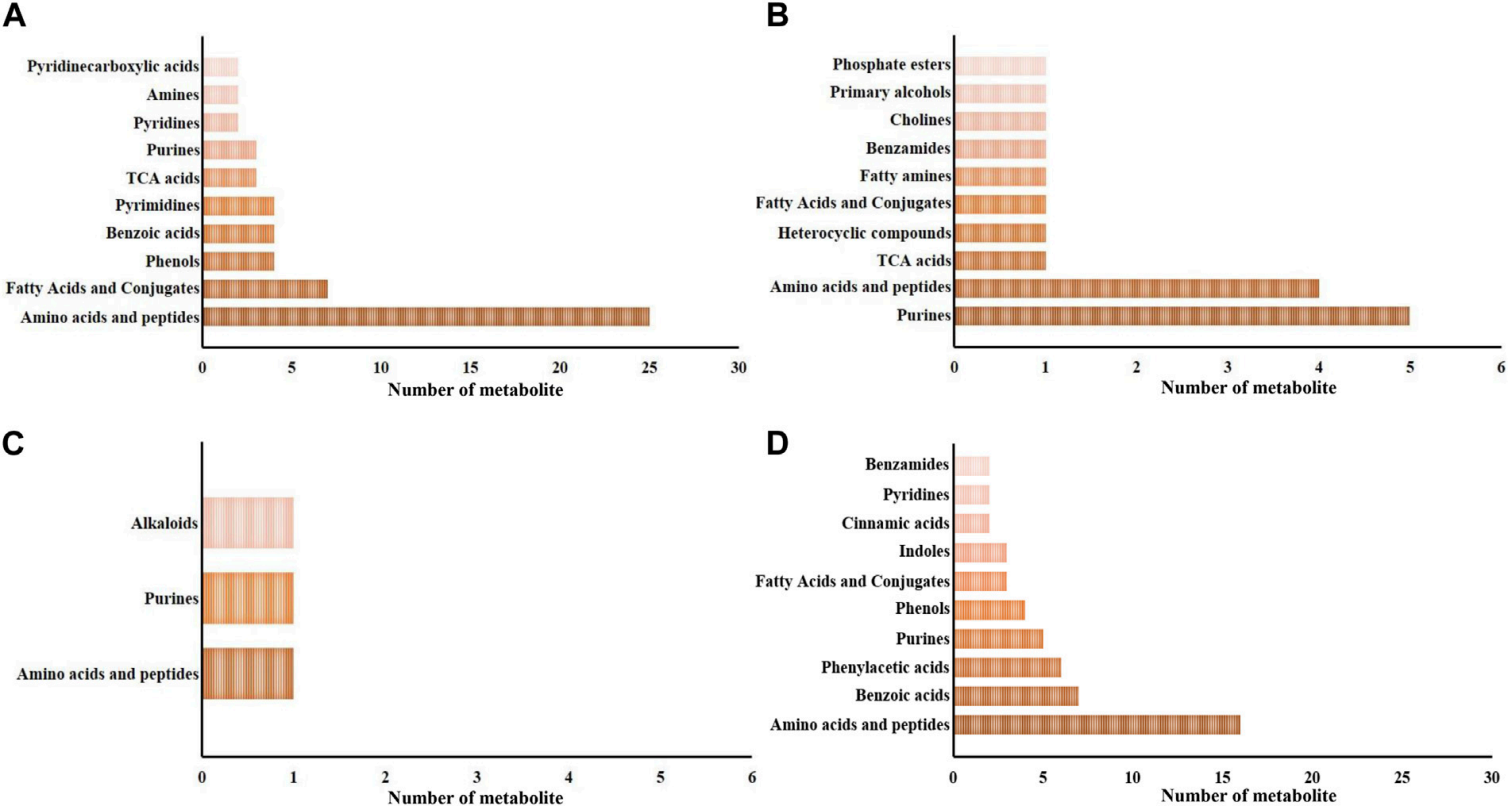
The major categories of the significantly changed in the epidermis **(A,B)** and gills **(C,D)** at 24 hpi **(A,C)** and 48 hpi **(B,D)**. Pre, the pre-pubertal molt group; Post, the post-pubertal molt group. *, significantly different (*p*-value < 0.05); **, very significantly different (*p*-value < 0.01); ***, extremely significantly different (*p*-value < 0.001).

Metabolites exhibiting significant differences between the SAI and DSI groups are detailed in the Supplementary Tables S1, S2. These encompass 98 and 20 metabolites from the epidermis at 24- and 48-h post-injection (hpi), respectively, and 4 and 79 metabolites from gills at the corresponding time points. At 24 hpi, the 98 significantly altered metabolites in the epidermis were distributed across 33 categories, with amino acids and peptides being the most predominant (25 out of 98, as shown in Figure 3A). At the 48 hpi mark, the 20 significantly altered metabolites fell into 10 categories, with purines being the most represented (5 out of 20, illustrated in Figure 3B).

Regarding gill-derived metabolomic profiles, the 4 significantly altered metabolites at 24 hpi were sorted into 3 categories (depicted in Figure 3C). In contrast, at 48 hpi, there were 79 significantly altered metabolites classified into 25 categories, with amino acids and peptides being the most enriched category (16 out of 79, displayed in Figure 3D).

### 3.3 Metabolite pathway analysis (MetPA)

In this investigation, Metabolite Pathway Analysis (MetPA) was utilized to discern significantly modulated pathways in the gills and epidermis following Pc-CHH gene expression silencing. Results indicated that at 24 h post-injection (hpi), 19 KEGG pathways in the epidermis were notably affected. The primary pathways impacted included amino acid metabolism and derivatives (6 out of 19), carbohydrate metabolism (5 out of 19), and cofactor and vitamin metabolism (4 out of 19, as detailed in Table 1). By 48 hpi, the number of significantly affected pathways had reduced to 6, suggesting a temporal association with CHH gene silencing and a subsequent decline in epidermal impact post-24 hpi (Table 1).

**TABLE 1:** MetPA of the significantly changed metabolites in the epidermis and gills of the crayfish *P. clarkii* at 24 and 48 hpi.

Tissue	Post-injection (h)	KEGG subclass	Pathway	Total compounds	Hits	*p-*value
Epidermis	24	Metabolism of cofactors and vitamins	Porphyrin and chlorophyll metabolism	24	3	0.00503
		Amino acid metabolism	Glycine, serine and threonine metabolism	30	12	0.00868
		Amino acid metabolism	Alanine, aspartate and glutamate metabolism	23	12	0.00992
		Carbohydrate metabolism	Glyoxylate and dicarboxylate metabolism	24	12	0.01544
		Carbohydrate metabolism	Ascorbate and aldarate metabolism	6	2	0.01817
		Amino acid metabolism	Arginine biosynthesis	12	9	0.01919
		Metabolism of other amino acids	beta-Alanine metabolism	14	7	0.02208
		Carbohydrate metabolism	Citrate cycle (TCA cycle)	20	9	0.02597
		Metabolism of cofactors and vitamins	Vitamin B6 metabolism	8	1	0.03051
		Translation	Aminoacyl-tRNA biosynthesis	48	19	0.03222
		Carbohydrate metabolism	Galactose metabolism	27	6	0.03346
		Metabolism of cofactors and vitamins	Thiamine metabolism	7	1	0.03848
		Metabolism of cofactors and vitamins	Biotin metabolism	10	2	0.04012
		Signal transduction	Phosphatidylinositol signaling system	28	1	0.04225
		Lipid metabolism	Glycerolipid metabolism	13	2	0.04393
		Amino acid metabolism	Phenylalanine, tyrosine and tryptophan biosynthesis	4	2	0.04483
		Carbohydrate metabolism	Amino sugar and nucleotide sugar metabolism	34	1	0.04577
		Nucleotide metabolism	Pyrimidine metabolism	40	12	0.04656
		Amino acid metabolism	Histidine metabolism	9	4	0.04900
	48	Signal transduction	Phosphatidylinositol signaling system	28	1	0.00515
		Biosynthesis of other secondary metabolites	Caffeine metabolism	10	1	0.00739
		Lipid metabolism	Sphingolipid metabolism	18	1	0.02807
		Carbohydrate metabolism	Butanoate metabolism	14	7	0.03327
		Metabolism of cofactors and vitamins	Biotin metabolism	10	2	0.04272
		Amino acid metabolism	D-Glutamine and D-glutamate metabolism	5	3	0.04611
Gills	24	-	-	-	-	-
	48	Lipid metabolism	Synthesis and degradation of ketone bodies	5	2	0.00086
		Biosynthesis of other secondary metabolites	Caffeine metabolism	10	1	0.00936
		Amino acid metabolism	Tryptophan metabolism	30	4	0.01111
		Amino acid metabolism	Tyrosine metabolism	33	10	0.01355
		Metabolism of cofactors and vitamins	Ubiquinone and other terpenoid-quinone biosynthesis	9	2	0.01687
		Amino acid metabolism	Cysteine and methionine metabolism	32	9	0.01974
		Amino acid metabolism	Phenylalanine metabolism	7	4	0.02119
		Metabolism of other amino acids	Taurine and hypotaurine metabolism	7	2	0.02364
		Amino acid metabolism	Glycine, serine and threonine metabolism	30	12	0.02790
		Lipid metabolism	Sphingolipid metabolism	18	1	0.02901
		Carbohydrate metabolism	Pyruvate metabolism	22	7	0.02944
		Metabolism of cofactors and vitamins	Nicotinate and nicotinamide metabolism	9	5	0.03564
		Carbohydrate metabolism	Glyoxylate and dicarboxylate metabolism	24	12	0.03835
		Carbohydrate metabolism	Citrate cycle (TCA cycle)	20	9	0.03879
		Signal transduction	Phosphatidylinositol signaling system	28	1	0.03939
		Lipid metabolism	Glycerophospholipid metabolism	32	5	0.04201

In gills, MetPA did not reveal any significantly modulated pathways at 24 hpi. However, at 48 hpi, 16 pathways were identified as significantly influenced—specifically, 6 related to amino acid metabolism and derivatives, 3 associated with lipid metabolism, and 3 with carbohydrate metabolism. The data implies that the effects of CHH gene silencing on gill metabolism may be delayed compared to the changes observed in CHH peptide levels in hemolymph (Li et al., 2017).

### 3.4 Metabolite set enrichment analysis (MSEA)

The Metabolite Set Enrichment Analysis (MSEA) revealed that CHH gene silencing significantly regulated 24 metabolite set categories in the epidermis at 24 hpi and 7 categories at 48 hpi. Of these, 17 at 24 hpi and 6 at 48 hpi were congruent with the Metabolite Pathway Analysis (MetPA) findings (Tables 1, 2). Conversely, in gills, MSEA identified 15 metabolite sets at 48 hpi, with 13 sets aligning with the MetPA results (Tables 1, 2).

**TABLE 2:** MSEA of the significantly changed metabolites in the epidermis and gills of the crayfish *P. clarkii* at 24 and 48 hpi.

Tissue	Post-injection (h)	Metabolite sets	Total compounds	Hits	*p*-value
Epidermis	24	Histidine metabolism	16	7	0.00359
		Selenocompound metabolism	20	1	0.00385
		Porphyrin and chlorophyll metabolism	30	3	0.00503
		Glycine, serine and threonine metabolism	33	15	0.00889
		Alanine, aspartate and glutamate metabolism	28	14	0.01011
		Ascorbate and aldarate metabolism	8	2	0.01817
		Nicotinate and nicotinamide metabolism	15	8	0.02095
		beta-Alanine metabolism	21	7	0.02208
		Starch and sucrose metabolism	18	3	0.02711
		Citrate cycle (TCA cycle)	20	8	0.02740
		Galactose metabolism	27	9	0.03079
		Glyoxylate and dicarboxylate metabolism	32	12	0.03206
		Aminoacyl-tRNA biosynthesis	48	19	0.03222
		Arginine biosynthesis	14	10	0.03352
		Phenylalanine metabolism	10	5	0.03459
		Thiamine metabolism	7	1	0.03848
		Biotin metabolism	10	2	0.04012
		Purine metabolism	65	17	0.04149
		Phosphatidylinositol signaling system	28	1	0.04225
		Tyrosine metabolism	42	11	0.04301
		Phenylalanine, tyrosine and tryptophan biosynthesis	4	2	0.04483
		Caffeine metabolism	10	2	0.04607
		Pyrimidine metabolism	39	12	0.04656
		Amino sugar and nucleotide sugar metabolism	37	4	0.04691
	48	Caffeine metabolism	10	2	0.00259
		Phosphatidylinositol signaling system	28	1	0.00515
		Sphingolipid metabolism	21	1	0.02807
		Butanoate metabolism	15	7	0.03808
		Biotin metabolism	10	2	0.04272
		D-Glutamine and D-glutamate metabolism	6	3	0.04611
		Synthesis and degradation of ketone bodies	5	1	0.04664
Gills	24	—	—	—	—
	48	Caffeine metabolism	10	2	0.00300
		Synthesis and degradation of ketone bodies	5	1	0.00449
		Tyrosine metabolism	42	11	0.01101
		Tryptophan metabolism	41	4	0.01111
		Cysteine and methionine metabolism	33	8	0.01456
		Ubiquinone and other terpenoid-quinone biosynthesis	9	2	0.01687
		Phenylalanine metabolism	10	5	0.01709
		Taurine and hypotaurine metabolism	8	2	0.02364
		Sphingolipid metabolism	21	1	0.02901
		Nicotinate and nicotinamide metabolism	15	8	0.03229
		Glycine, serine and threonine metabolism	33	15	0.03257
		Phosphatidylinositol signaling system	28	1	0.03939
		Glycerophospholipid metabolism	36	5	0.04201
		Selenocompound metabolism	20	1	0.04761
		Histidine metabolism	16	7	0.04834

Notably, MSEA demonstrated significant regulation of selenocompound metabolism in the epidermis at 24 hpi, along with nicotinate and nicotinamide metabolism, and starch and sucrose metabolism (Table 2). Concurrently, MetPA highlighted the substantial influence on vitamin B6 metabolism and glycerolipid metabolism at the same time point (Table 1). MSEA also indicated significant effects on the synthesis and degradation of ketone bodies at 48 hpi (Table 2), suggesting a marked impact on lipid metabolism in the epidermis following CHH gene silencing. In gills, MSEA identified selenocompound and histidine metabolism as significantly regulated, while MetPA confirmed notable modulation of pyruvate metabolism, glyoxylate and dicarboxylate metabolism, and the citrate cycle (Table 1). A synergistic evaluation using both MetPA and MSEA may afford a more holistic understanding of the physiological alterations in both epidermis and gills post gene silencing.

### 3.5 Significantly changed metabolites involved in energy metabolism

**FIGURE 4:**
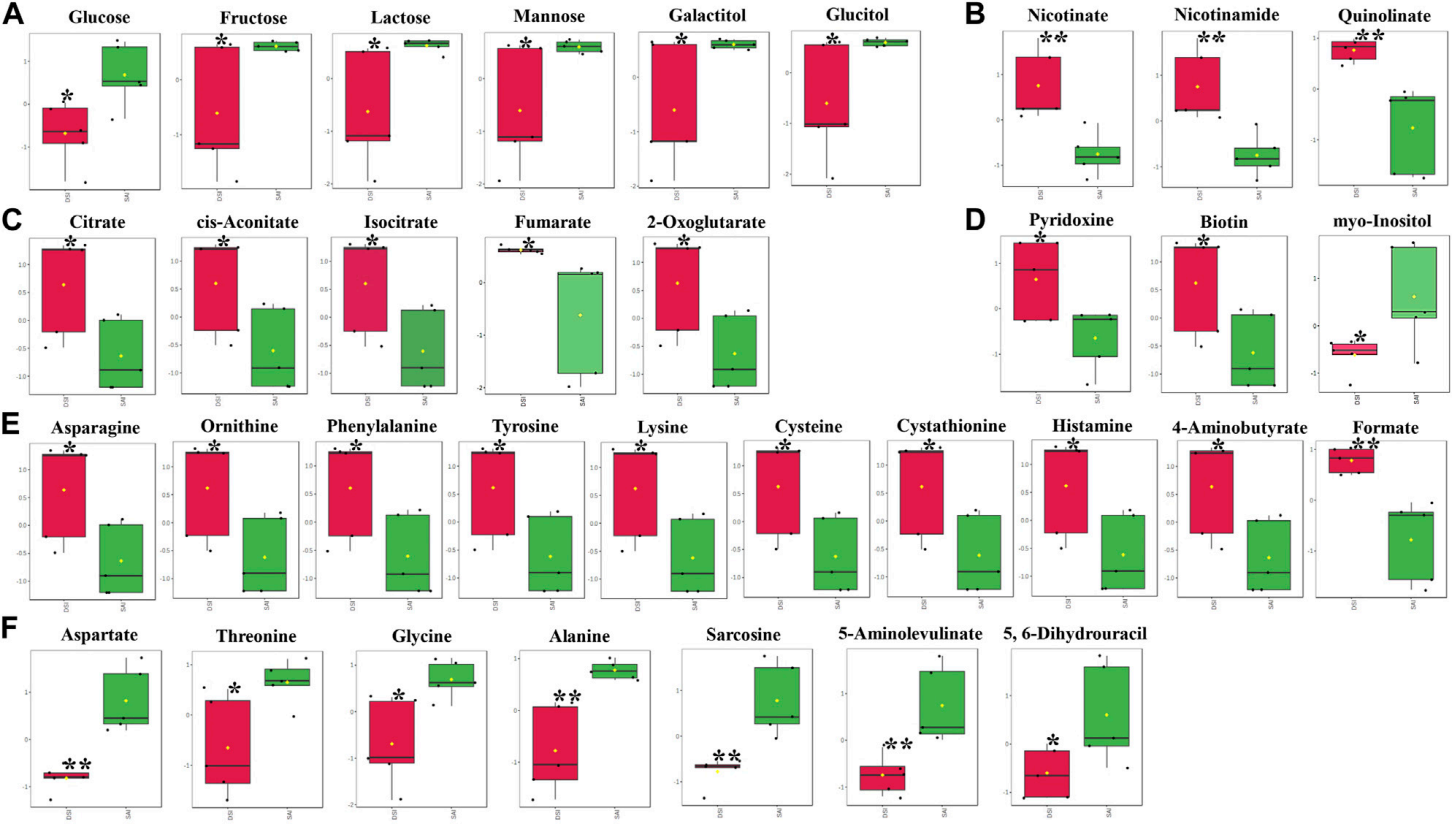
The significantly changed metabolites in the epidermis at 24 hpi. *, significantly different (*p*-value < 0.05); **, very significantly different (*p*-value < 0.01); ***, extremely significantly different (*p*-value < 0.001). **(A)**, the significantly changed metabolites associated with the carbohydrate metabolism; **(B)**, the significantly changed components in nicotinate and nicotinamide metabolism; **(C)**, the significantly changed metabolites involved in the citrate cycle; **(D)**, the significantly changed vitamins; **(E)**, the significantly increased amino acids; **(F)**, the significantly decreased amino acids.

At 24 hpi, the levels of most metabolites associated with carbohydrate metabolism were significantly reduced in the epidermis, including glucose, fructose, lactose, mannose, galactitol, and glucitol (Figure 4A). In contrast, metabolites involved in the citrate cycle exhibited a significant increase (Figure 4C). Concurrently, levels of nicotinate, nicotinamide, and quinolinate, key components in Nicotinate and Nicotinamide metabolism, markedly increased following CHH gene suppression (Figure 4B). This study also identified fluctuations in three vitamins: pyridoxine and biotin levels increased notably, while myo-inositol decreased (Figure 4D). Additionally, amino acids and vitamins underwent significant regulation: 17 amino acids altered in concentration, with 10 showing an increase (Figure 4E) and 7 showing a decrease (Figure 4F) after CHH gene silencing.

**FIGURE 5:**
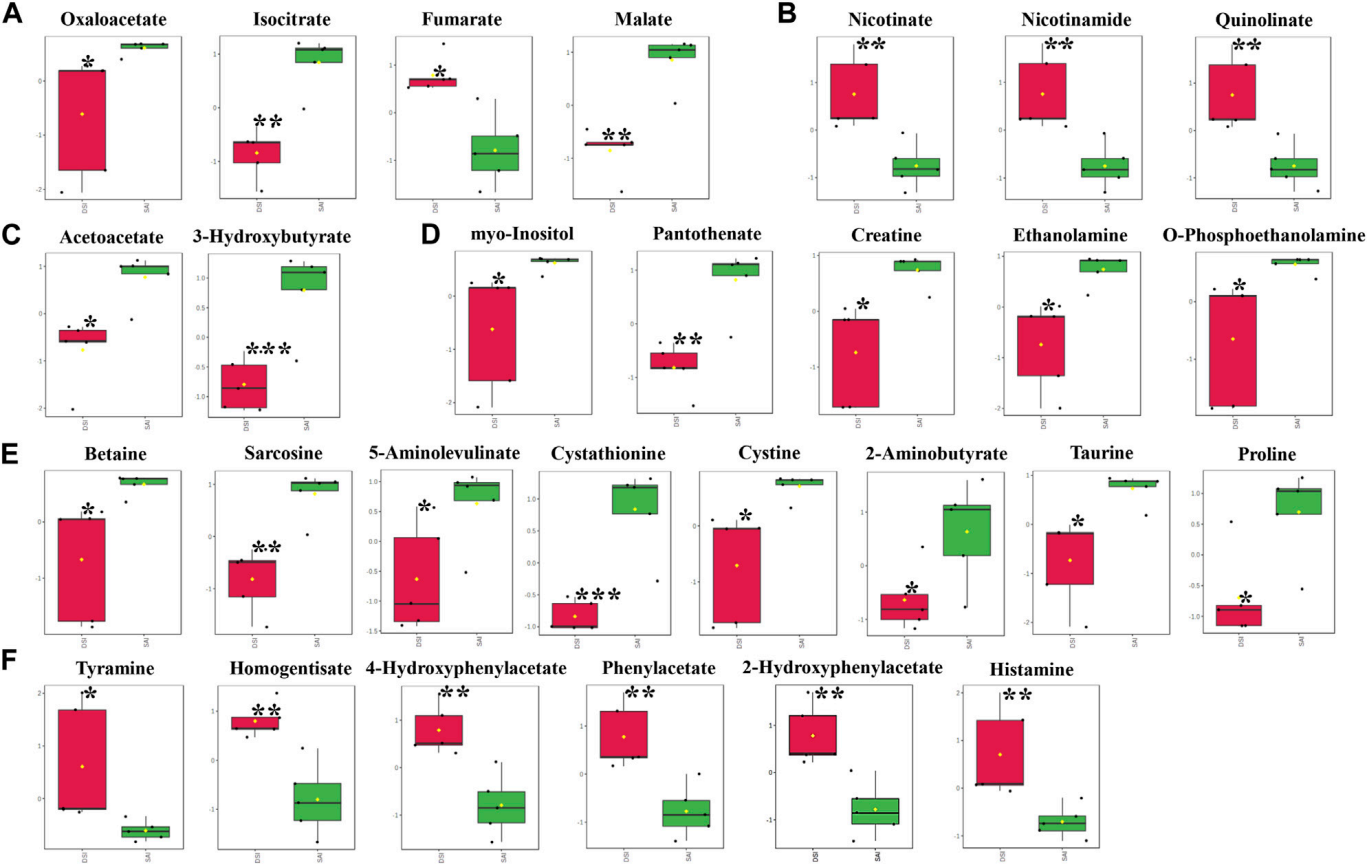
The significantly changed metabolites in the gills at 48 hpi. *, significantly different (*p*-value < 0.05); **, very significantly different (*p*-value < 0.01); ***, extremely significantly different (*p*-value < 0.001). **(A)**, the significantly changed metabolites associated with the Tricarboxylic Acid (TCA) cycle; **(B)**, the significantly changed components in nicotinate and nicotinamide metabolism; **(C)**, the significantly changed ketone bodies, including acetoacetate and 3-hydroxybutyrate; **(D)**, the significantly changed vitamins or derivatives; **(E)**, the significantly declined amino acids; **(F)**, the significantly increased amino acids.

In the gills, at 48 hpi, there was a significant decrease in the principal metabolites involved in the Tricarboxylic Acid (TCA) cycle, including oxaloacetate, isocitrate, fumarate, and malate (Figure 5A). Similar to the epidermis at 24 hpi, the levels of nicotinate, nicotinamide, and quinolinate were significantly elevated in the gills at 48 hpi (Figure 5B). Ketone bodies, acetoacetate and 3-hydroxybutyrate, produced by the hepatopancreas, were significantly reduced in the gills, suggesting an upregulation of lipid metabolism for energy production (Figure 5C). Furthermore, all five identified vitamins or derivatives in gills were significantly diminished in concentration (Figure 5D). The study also revealed a substantial decline in 14 amino acids in the gills at 48 hpi, comprising 8 decreasing amino acids and derivatives, and 6 increasing amino acids or derivatives (Figures 5E, F).

### 3.6 Receiver operating characteristics (ROC) analysis

**FIGURE 6:**
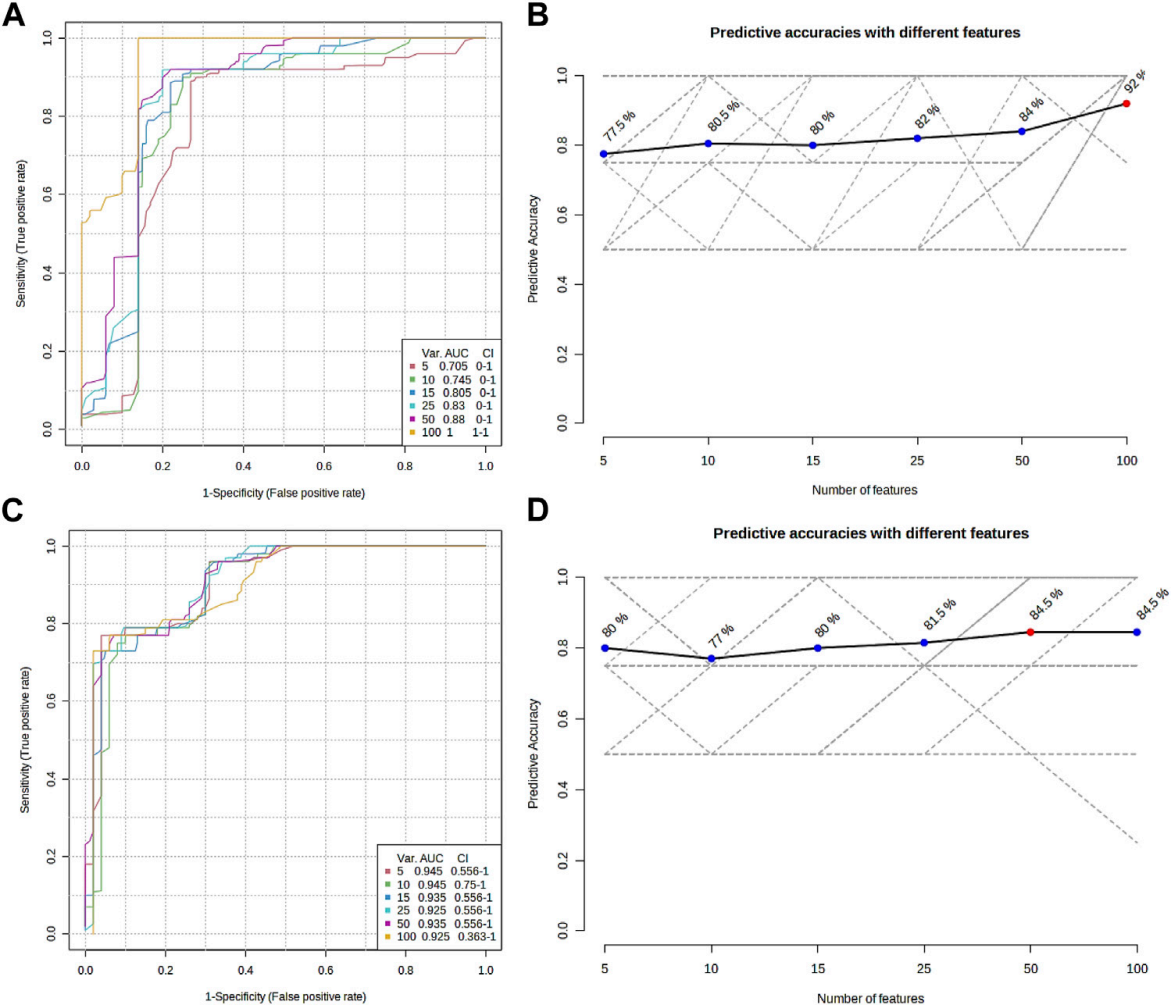
Overview of the multiple ROC analyses on the metabolomic profile from the epidermis **(A,B)** and gills **(C,D)** of *P. clarkii*. **(A,C)**, multiple ROC curves; **(B,D)**, predictive accuracies with different features.

The metabolomic profiling of the epidermis at 24 h post-injection (hpi) revealed through classical univariate Receiver Operating Characteristic (ROC) analysis that 51 metabolites achieved a perfect Area Under the Curve (AUC) of 1.0. Additionally, multivariate ROC analysis, employing cross-validation, demonstrated that models incorporating over 100 metabolites attained the highest AUC (1.0, illustrated in Figure 6A), while models with exactly 100 metabolites reached an optimal predictive accuracy of 92% (depicted in Figure 6B).

From the metabolomic profiling of the gills at 48 hpi, the univariate ROC analysis identified 117 metabolites each with an AUC of 1.0. The multivariate ROC analysis further showed that models with more than 10 metabolites yielded the highest AUC (0.945, as shown in Figure 6C), and the model with a minimum of 50 metabolites achieved the greatest predictive accuracy (84.5%, presented in Figure 6D).

The combined univariate and multivariate ROC curve analyses pinpointed the top 10 metabolites from the epidermis and gills as the most predictive biomarkers, selected based on their highest frequency of selection and average importance within the biomarker panel (refer to Table 3; Supplementary Figures S1, S2). It should be noted that no potential biomarkers were discerned from the metabolomic profiles of the epidermis at 48 hpi or the gills at 24 hpi.

**TABLE 3:** The potential biomarkers in epidermis at 24 hpi and in gills at 48 hpi.

Tissue	Post-injection (h)	Metabolite	AUC	*p*-value	Fold change	Rank frequency	Importance	DSI	SAI
Epidermis	24	Nicotinate	1	0.00423	−0.03721	1.00	1.50499	High	Low
		Adenine	1	0.00434	−0.03884	1.00	1.50140	High	Low
		Niacinamide	1	0.00606	−0.04223	0.92	1.50117	High	Low
		Phthalate	1	0.00949	−0.04964	0.92	1.49745	High	Low
		Quinolinate	1	0.00571	−0.04559	0.92	1.49690	High	Low
		4-Hydroxybenzoate	1	0.01553	−0.05510	0.92	1.49519	High	Low
		Cinnamate	1	0.01218	−0.05531	0.92	1.49330	High	Low
		Kynurenate	1	0.00851	−0.05071	0.92	1.49317	High	Low
		Urocanate	1	0.01205	−0.05549	0.92	1.49135	High	Low
		Imidazole	1	0.00901	−0.05340	0.92	1.49067	High	Low
	48	—	—	—	—	—	—	—	—
Gills	24	—	—	—	—	—	—	—	—
	48	Cystathionine	1	0.00031	2.2896	1	1.53669	Low	High
		3-Hydroxybutyrate	1	0.00039	2.2871	0.94	1.67259	Low	High
		4-Hydroxyphenylacetate	1	0.00204	2.622	0.92	1.41440	High	Low
		3-Hydroxyphenylacetate	1	0.00201	2.668	0.92	1.39914	High	Low
		5-Methoxysalicylate	1	0.00204	2.6375	0.92	1.39296	High	Low
		2-Hydroxyphenylacetate	1	0.00220	2.6882	0.92	1.37471	High	Low
		Phenylacetate	1	0.00236	2.6826	0.92	1.36413	High	Low
		Gentisate	1	0.00294	2.7815	0.92	1.33337	High	Low
		5-Hydroxyindole-3-acetate	1	0.00205	2.622	0.9	1.42663	High	Low
		Phenylacetylglycine	1	0.00182	2.6106	0.88	1.41738	High	Low

## 4 Discussion

In this study, we leveraged a previously established RNA interference (RNAi) system to explore the tissue-specific impacts of the peptide Pc-CHH on the gills and epidermis of the crayfish *Procambarus clarkii*, in tandem with metabolomic profiling. With RNAi effectively diminishing Pc-CHH peptide levels in the hemolymph at both 24 and 48 h post-injection (hpi) as reported by Li et al. (2017), we correspondingly collected epidermis and gill samples at these intervals. Metabolomic analysis utilizing ^1^H NMR spectroscopy identified a total of 269 metabolites across the sampled tissues, comprising 98 (36.43%) from the epidermis at 24 hpi, 20 (7.43%) at 48 hpi, and in the gills, 4 (1.49%) at 24 hpi, and 79 (29.37%) at 48 hpi. The influence of CHH on the epidermis was immediate, mirroring the fluctuations of CHH peptides in the hemolymph, with a peak at 24 hpi and a decline by 48 hpi. Conversely, the effect on the gills manifested with a delay, becoming pronounced at 48 hpi. These findings suggest that Pc-CHH’s impact varies across different tissues and organs, including the hepatopancreas and muscle as previously noted by Li et al. (2017), in addition to the epidermis and gills examined in this study.

Utilizing various enrichment methodologies, both Metabolomic Pathway Analysis (MetPA) and Metabolite Set Enrichment Analysis (MSEA) identified similarly significant modulated pathways (metabolite sets) within the epidermis and gills. At 24 h post-injection (hpi), 17 pathways or metabolite sets in the epidermis were unanimously identified (as outlined in Tables 1, 2), primarily encompassing metabolism of amino acids (and derivatives) (35.29%), carbohydrate metabolism (29.41%), and metabolism of cofactors and vitamins (17.65%). These pathways represent the central metabolic processes influenced by CHH silencing in the epidermis. At 48 hpi, 13 consistent pathways or metabolite sets were observed in the epidermis (referenced in Tables 1, 2), prominently involved in the metabolism of amino acids (and derivatives) (46.15%), lipid metabolism (23.08%), and metabolism of cofactors and vitamins (15.38%).

Collectively, these findings indicate that amino acid metabolism (and related processes) was the most extensively affected category in both the epidermis and gills. Additionally, while carbohydrate metabolism was profoundly altered in the epidermis, lipid metabolism experienced significant modulation in the gills at the respective time points. This divergence may imply that the primary energy substrates differ between the epidermis and gills. MetPA further highlighted the substantial regulation of glycerolipid metabolism in the epidermis at 24 hpi, and carbohydrate metabolism in the gills at 48 hpi—including pyruvate metabolism, glyoxylate and dicarboxylate metabolism, and the citrate cycle—suggesting that the energy sources in both tissues are not uniform, but rather vary and are specific to the organ or tissue.

In the epidermis of *P. clarkii*, levels of carbohydrates such as glucose, fructose, lactose, mannose, galactitol, and glucitol (Figure 4A) were significantly reduced, likely due to the marked decrease of Pc-CHH peptide levels in the hemolymph. CHH is known to play a pivotal role in the regulation of carbohydrate metabolism: a reduction of CHH peptide levels in the hemolymph corresponds with a decreased carbohydrate influx from the hemolymph (Nagai et al., 2011; Li et al., 2017; Liu et al., 2019; Chen et al., 2020). Additionally, TCA cycle intermediates, including citrate, cis-aconitate, isocitrate, fumarate, and 2-oxoglutarate (Figure 4C), exhibited a notable increase, suggesting an upregulation of the TCA cycle which could deplete upstream substrates and further accentuate the reduction in carbohydrate concentrations.

Conversely, carbohydrate levels in the gills did not exhibit a significant reduction at either 24 or 48 hpi (Figures 3, 5), indicating that the gill carbohydrate supply was unaffected by CHH gene silencing. The marked decline in oxaloacetate, isocitrate, and malate levels suggests a potential slowdown in upstream metabolism (e.g., pyruvate production), while the significant increase in fumarate levels implies an alternative substrate source for the TCA cycle. Meanwhile, the notable decrease in ketone bodies in the gills at 48 hpi, including acetoacetate and 3-hydroxybutyrate, indicates a lipid-based energy supply has been recruited and the lipid metabolism has been upregulated in gills during CHH gene silencing. Given the gills’ crucial role in ionic transport and osmoregulation (Henry et al., 2012), a consistent and diverse energy supply is essential.

Nicotinate and nicotinamide metabolism, which involves the metabolism of NAD^+^ and NADP^+^ coenzymes and has varied metabolic implications (Li et al., 2017), was identified as significantly modulated in the epidermis at 24 hpi by MetPA, and in the gills at 48 hpi by both MetPA and MSEA. The significant elevation in the levels of nicotinate, nicotinamide, and quinolinate in both the epidermis at 24 hpi and the gills at 48 hpi suggests an upregulation of nicotinate and nicotinamide metabolism, ensuring ample NAD^+^ flux. However, the elevated NAD^+^ may be utilized differently in the epidermis and gills: in the epidermis, it likely supports the enhanced TCA cycle for energy production, while in the gills, it may be involved in regulating lipid metabolism through the formation of NADP^+^ (Rey et al., 2016).

At 24 hpi in the epidermis, there was a significant increase in the levels of 10 amino acids (or their derivatives), while the content of another 7 amino acids (or their derivatives) decreased markedly (Figures 4E, F). Conversely, 8 amino acids (or their derivatives) showed a clear decline in concentration, whereas the levels of another 6 amino acids (or their derivatives) experienced a notable rise (Figures 5E, F). Given that both the epidermis and gills are located on the body surface, they may be implicated in osmoregulation and molting. Nonetheless, the distinct metabolic profiles of amino acids between these two tissues suggest their differing predominant functions following CHH gene expression silencing. Considering the significant modulation of amino sugar and nucleotide sugar metabolism, the epidermis appears to play vital roles in maintaining cuticular integrity or in osmoregulation through the preservation of the carapace. In contrast, the gills seem to engage more directly in osmoregulation via active ion transport or Na^+^-K^+^-ATPase activity, which necessitates adequate energy supply.

## 5 Conclusion

In conclusion, leveraging the previously established RNA interference system targeting the CHH dsRNA, we conducted an extensive metabolomic analysis of the gills and epidermis of *P. clarkii*. A total of 269 metabolites were identified within both tissues. These metabolites spanned several major categories, including amino acids and peptides, fatty acids and their conjugates, purines, monosaccharides, and organic dicarboxylic acids. Notably, 98 metabolites in the epidermis at 24 hpi and 79 in the gills at 48 hpi exhibited significant alterations. These changes were most prominent in the metabolism of amino acids, carbohydrates, lipids, and cofactors and vitamins. The study revealed tissue/organ-specific metabolic responses: in the epidermis, the effects of CHH gene silencing paralleled the gene knockdown, while in the gills, responses were delayed. The data suggest diverse energy substrates, with ketone bodies likely serving as the primary energy source in gills, in contrast to carbohydrates in the epidermis. Nicotinate and nicotinamide metabolism were markedly stimulated in both tissues, though they may participate in different physiological functions. In the epidermis, there was a clear increase in major amino acids, whereas in the gills, a decrease was observed, aligning with their respective tissue/organ-specific roles. This investigation lays the groundwork for future studies aimed at elucidating the regulatory mechanisms of energy metabolism, osmoregulation, and molting in the epidermis and gills of *P. clarkii*.

## Data Availability

The original contributions presented in the study are included in the article/Supplementary Material, further inquiries can be directed to the corresponding author.
