# Supplementary material for: Silencing of crustacean hyperglycemic hormone gene expression reveals the characteristic energy and metabolic changes in the gills and epidermis of crayfish Procambarus clarkii
Source: Front Physiol. 2024 Jan 10;14:1349106. doi: 10.3389/fphys.2023.1349106 (PMC10806126; doi:10.3389/fphys.2023.1349106)
Supplement: Supplementary file 1 [file DataSheet1.DOCX]

Supplementary Material

**Supplementary table 1** The significantly changed metabolites in the epidermis at 24 and 48 hpi.

|  | Metabolite | Saline-injection group (SAI) | | CHH dsRNA injection group (DSI) | | *P*-value |
| --- | --- | --- | --- | --- | --- | --- |
|  |  | Mean | Std | Mean | Std |  |
| 24 hpi | 1-Methylnicotinamide | 0.00436 | 0.00242 | 0.00370 | 0.00170 | 0.02323 |
|  | 2'-Deoxyguanosine | 0.04935 | 0.01491 | 0.02430 | 0.01317 | 0.02837 |
|  | 2-Ethylacrylate | 0.01521 | 0.00721 | 0.01135 | 0.00321 | 0.01809 |
|  | 2-Hydroxy-3-methylvalerate | 0.01737 | 0.00289 | 0.01305 | 0.00216 | 0.02460 |
|  | 2-Oxoglutarate | 1.02416 | 0.05164 | 1.01917 | 0.03058 | 0.01447 |
|  | 3,5-Dibromotyrosine | 2.07007 | 0.06880 | 2.07184 | 0.04375 | 0.01673 |
|  | 3-Aminoisobutyrate | 2.06627 | 0.04451 | 2.07441 | 0.02966 | 0.01861 |
|  | 3-Chlorotyrosine | 2.07071 | 0.05880 | 2.07599 | 0.03877 | 0.01738 |
|  | 3-Hydromuconate | 2.06223 | 0.06801 | 2.06891 | 0.04164 | 0.01659 |
|  | 3-Indoxylsulfate | 0.00498 | 0.00300 | 0.00478 | 0.00177 | 0.01264 |
|  | 3-Methyl-2-oxovalerate | 0.02055 | 0.00505 | 0.00938 | 0.00442 | 0.00043 |
|  | 3-Methylxanthine | 0.01128 | 0.00403 | 0.00742 | 0.00347 | 0.03167 |
|  | 3-Phenyllactate | 1.04940 | 0.01396 | 1.04437 | 0.01463 | 0.02101 |
|  | 3-Phenylpropionate | 0.07613 | 0.02139 | 0.03614 | 0.01858 | 0.00585 |
|  | 4-Aminobutyrate | 1.98850 | 0.11712 | 1.96923 | 0.07921 | 0.01311 |
|  | 4-Hydroxybenzoate | 0.00835 | 0.00517 | 0.00803 | 0.00303 | 0.01553 |
|  | 4-Hydroxyphenyllactate | 1.05172 | 0.04243 | 1.04498 | 0.02753 | 0.01617 |
|  | 5,6-Dihydrouracil | 0.02103 | 0.00365 | 0.00981 | 0.00334 | 0.03545 |
|  | 5-Aminolevulinate | 0.06984 | 0.01395 | 0.03176 | 0.01183 | 0.00195 |
|  | 5-Hydroxylysine | 2.15247 | 0.04709 | 2.11050 | 0.04135 | 0.02136 |
|  | Acetamide | 0.00676 | 0.00364 | 0.00529 | 0.00151 | 0.03189 |
|  | Acetaminophen | 0.01419 | 0.00862 | 0.01264 | 0.00462 | 0.02287 |
|  | Acetoacetate | 0.00647 | 0.00117 | 0.00302 | 0.00106 | 0.04146 |
|  | Acetylsalicylate | 0.00763 | 0.00330 | 0.00635 | 0.00170 | 0.00203 |
|  | Adenine | 0.00563 | 0.00306 | 0.00548 | 0.00186 | 0.00434 |
|  | Alanine | 0.00508 | 0.00144 | 0.00214 | 0.00141 | 0.01343 |
|  | Allantoin | 0.00552 | 0.00387 | 0.00513 | 0.00223 | 0.04403 |
|  | Anserine | 3.11921 | 0.01956 | 3.12618 | 0.01130 | 0.02314 |
|  | Asparagine | 2.12543 | 0.15074 | 2.07956 | 0.09339 | 0.01249 |
|  | Aspartate | 0.03843 | 0.00844 | 0.01725 | 0.00774 | 0.00082 |
|  | Benzoate | 0.00710 | 0.00405 | 0.00682 | 0.00240 | 0.00756 |
|  | Biotin | 1.06311 | 0.05744 | 1.04207 | 0.03151 | 0.01592 |
|  | Cadaverine | 3.09346 | 0.20965 | 3.06249 | 0.14125 | 0.01157 |
|  | Caprylate | 0.03264 | 0.00721 | 0.02740 | 0.00234 | 0.01285 |
|  | Carnosine | 4.08848 | 0.02650 | 4.12798 | 0.00810 | 0.02311 |
|  | Cinnamate | 0.01132 | 0.00682 | 0.01089 | 0.00397 | 0.01219 |
|  | Citrate | 1.05528 | 0.07621 | 1.03516 | 0.04366 | 0.01243 |
|  | Citrulline | 2.04202 | 0.01856 | 2.06095 | 0.00539 | 0.02384 |
|  | Creatine | 1.01210 | 0.03886 | 1.01460 | 0.02459 | 0.01558 |
|  | Creatinine | 1.02202 | 0.00684 | 1.03017 | 0.00776 | 0.02095 |
|  | Cystathionine | 2.09374 | 0.05863 | 2.08327 | 0.03942 | 0.01843 |
|  | Cysteine | 2.06846 | 0.08447 | 2.06575 | 0.05268 | 0.01560 |
|  | Cystine | 1.06706 | 0.02110 | 1.05426 | 0.01691 | 0.02109 |
|  | Cytosine | 0.00466 | 0.00321 | 0.00437 | 0.00184 | 0.03808 |
|  | Ethanol | 0.01171 | 0.00226 | 0.00978 | 0.00213 | 0.00127 |
|  | Ethanolamine | 2.03011 | 0.01104 | 2.05123 | 0.00613 | 0.02243 |
|  | Ethylmalonate | 1.03240 | 0.01954 | 1.03716 | 0.01288 | 0.01909 |
|  | Ferulate | 0.00995 | 0.00580 | 0.00815 | 0.00392 | 0.04400 |
|  | Glucose | 0.06186 | 0.01486 | 0.02925 | 0.01648 | 0.04111 |
|  | Glutathione | 1.34117 | 0.14286 | 1.26399 | 0.14378 | 0.00722 |
|  | Glycylproline | 0.09666 | 0.02539 | 0.04383 | 0.02381 | 0.00594 |
|  | Hippurate | 0.01018 | 0.00535 | 0.00794 | 0.00401 | 0.04609 |
|  | Histamine | 1.05038 | 0.03161 | 1.04984 | 0.02065 | 0.01746 |
|  | Histidine | 1.07337 | 0.01135 | 1.06623 | 0.00465 | 0.02423 |
|  | Homocitrulline | 1.01664 | 0.00990 | 1.02368 | 0.00461 | 0.02466 |
|  | Homocystine | 0.03881 | 0.00819 | 0.01762 | 0.00652 | 0.00080 |
|  | Homoserine | 0.02501 | 0.00826 | 0.01071 | 0.00728 | 0.02132 |
|  | Imidazole | 0.01673 | 0.00973 | 0.01612 | 0.00572 | 0.00901 |
|  | Isocitrate | 1.06110 | 0.02067 | 1.05049 | 0.01580 | 0.02084 |
|  | Isopropanol | 0.01095 | 0.00271 | 0.00932 | 0.00245 | 0.00008 |
|  | Kynurenate | 0.00525 | 0.00304 | 0.00507 | 0.00179 | 0.00851 |
|  | Lysine | 2.07331 | 0.07752 | 2.06865 | 0.04595 | 0.01654 |
|  | Mandelate | 0.00424 | 0.00276 | 0.00381 | 0.00164 | 0.03595 |
|  | Methionine | 0.02955 | 0.00634 | 0.01354 | 0.00366 | 0.02620 |
|  | Methylguanidine | 0.00722 | 0.00145 | 0.00328 | 0.00130 | 0.00178 |
|  | N,N-Dimethylformamide | 1.01399 | 0.06049 | 1.00678 | 0.03725 | 0.01297 |
|  | N,N-Dimethylglycine | 0.01867 | 0.00498 | 0.00819 | 0.00503 | 0.01248 |
|  | N-Acetyltyrosine | 1.03317 | 0.00846 | 1.03648 | 0.00876 | 0.02137 |
|  | N-Carbamoylaspartate | 0.03341 | 0.00872 | 0.01756 | 0.00618 | 0.01807 |
|  | Niacinamide | 0.01522 | 0.00854 | 0.01478 | 0.00509 | 0.00606 |
|  | Nicotinate | 0.01286 | 0.00698 | 0.01253 | 0.00424 | 0.00423 |
|  | Ornithine | 1.04814 | 0.03907 | 1.04030 | 0.02409 | 0.01711 |
|  | Phenol | 0.00591 | 0.00378 | 0.00566 | 0.00219 | 0.02067 |
|  | Phenylalanine | 1.05920 | 0.02044 | 1.05328 | 0.01455 | 0.02047 |
|  | Phthalate | 0.01005 | 0.00590 | 0.00971 | 0.00344 | 0.00949 |
|  | Pipecolate | 0.96681 | 0.07592 | 0.94082 | 0.05971 | 0.01093 |
|  | Protocatechuate | 0.00878 | 0.00542 | 0.00840 | 0.00315 | 0.01563 |
|  | Putrescine | 1.02593 | 0.02769 | 1.03053 | 0.01750 | 0.01753 |
|  | Pyridoxine | 0.02364 | 0.00887 | 0.01551 | 0.00525 | 0.01199 |
|  | Quinolinate | 0.00981 | 0.00546 | 0.00951 | 0.00325 | 0.00571 |
|  | S-Adenosylhomocysteine | 2.14223 | 0.08126 | 2.10999 | 0.05336 | 0.01776 |
|  | S-Sulfocysteine | 0.03228 | 0.00919 | 0.01429 | 0.01081 | 0.04782 |
|  | Sarcosine | 0.00577 | 0.00121 | 0.00262 | 0.00119 | 0.00342 |
|  | Succinylacetone | 0.03472 | 0.00733 | 0.01564 | 0.00615 | 0.00058 |
|  | Thymidine | 0.00877 | 0.00257 | 0.00437 | 0.00204 | 0.00370 |
|  | Thymol | 1.03150 | 0.00406 | 1.04249 | 0.00306 | 0.02233 |
|  | Trigonelline | 0.00539 | 0.00298 | 0.00462 | 0.00208 | 0.02018 |
|  | Trimethylamine | 0.00894 | 0.00214 | 0.00399 | 0.00200 | 0.00154 |
|  | Tyramine | 0.05762 | 0.01583 | 0.02928 | 0.01497 | 0.04662 |
|  | Tyrosine | 2.06566 | 0.04941 | 2.07252 | 0.03448 | 0.01807 |
|  | Urocanate | 0.01666 | 0.01001 | 0.01603 | 0.00587 | 0.01205 |
|  | cis-Aconitate | 1.02487 | 0.00344 | 1.03681 | 0.00332 | 0.02205 |
|  | dCTP | 0.01623 | 0.00633 | 0.01066 | 0.00405 | 0.02014 |
|  | o-Cresol | 0.00838 | 0.00414 | 0.00685 | 0.00203 | 0.00932 |
|  | p-Cresol | 0.01405 | 0.00775 | 0.01190 | 0.00397 | 0.01613 |
|  | Gamma-Glutamylphenylalanine | 0.09678 | 0.02368 | 0.04907 | 0.01892 | 0.00876 |
|  | Pi-Methylhistidine | 2.08212 | 0.02212 | 2.08799 | 0.02331 | 0.02056 |
|  | Tau-Methylhistidine | 2.05242 | 0.00764 | 2.06995 | 0.00872 | 0.02223 |
| 48 hpi | 1,3-Dimethylurate | 0.00603 | 0.00213 | 0.01535 | 0.00435 | 0.01785 |
|  | 1,7-Dimethylxanthine | 0.00612 | 0.00172 | 0.01406 | 0.00502 | 0.01062 |
|  | 2-Deoxyadenosine | 1.02550 | 1.00885 | 0.04055 | 0.01793 | 0.04791 |
|  | 2-Oxoglutarate | 0.21129 | 0.43656 | 1.03115 | 0.06377 | 0.02146 |
|  | 4-Aminobutyrate | 0.41245 | 0.84382 | 1.65725 | 0.54190 | 0.02381 |
|  | 5-Hydroxylysine | 2.93185 | 1.15153 | 2.17033 | 0.07069 | 0.03774 |
|  | Biotin | 0.23281 | 0.43820 | 1.07507 | 0.07101 | 0.02231 |
|  | Cadaverine | 0.62399 | 1.31306 | 2.69792 | 0.69487 | 0.02678 |
|  | Caffeine | 0.00808 | 0.00562 | 0.01398 | 0.00511 | 0.01890 |
|  | Choline | 0.01354 | 0.00875 | 0.02695 | 0.00745 | 0.02124 |
|  | Creatine | 0.20882 | 0.43811 | 1.02359 | 0.05166 | 0.01698 |
|  | Glutathione | 2.15797 | 1.00393 | 1.30150 | 0.23223 | 0.01069 |
|  | Methanol | 0.00223 | 0.00080 | 0.00611 | 0.00169 | 0.02470 |
|  | O-Phosphoethanolamine | 0.02222 | 0.01496 | 0.05000 | 0.01017 | 0.03774 |
|  | Theophylline | 0.01057 | 0.00459 | 0.01803 | 0.00687 | 0.02100 |
|  | Threonine | 0.01215 | 0.00738 | 0.02045 | 0.00853 | 0.02819 |
|  | Trimethylamine N-oxide | 0.00695 | 0.00248 | 0.01746 | 0.00483 | 0.01712 |
|  | Tryptophan | 0.02526 | 0.01381 | 0.04922 | 0.01447 | 0.02644 |
|  | myo-Inositol | 0.01196 | 0.00721 | 0.02184 | 0.00704 | 0.01093 |
|  | trans-4-Hydroxy-L-proline | 0.01949 | 0.00751 | 0.03865 | 0.01241 | 0.02494 |

**Supplementary table 2** The significantly changed metabolites in the gills at 24 and 48 hpi.

|  | Metabolite | Saline-injection group (SAI) | | CHH dsRNA injection group (DSI) | | *P*-value |
| --- | --- | --- | --- | --- | --- | --- |
|  |  | Mean | Std | Mean | Std |  |
| 24 hpi | ADP | 0.57911 | 0.28141 | 0.22670 | 0.11936 | 0.04479 |
|  | Carnosine | 0.10133 | 0.10457 | 0.14458 | 0.07801 | 0.04499 |
|  | N-Acetyltyrosine | 0.02310 | 0.02514 | 0.03416 | 0.01869 | 0.04576 |
|  | Trigonelline | 0.00624 | 0.00597 | 0.00753 | 0.00393 | 0.04991 |
| 48 hpi | 1,3-Dimethylurate | 0.02648 | 0.01430 | 0.13015 | 0.15677 | 0.03067 |
|  | 2-Hydroxyisovalerate | 0.05429 | 0.02805 | 0.26447 | 0.31846 | 0.03551 |
|  | 2-Hydroxyphenylacetate | 0.03041 | 0.02226 | 0.19602 | 0.23819 | 0.00220 |
|  | 2-Octenoate | 0.06171 | 0.03626 | 0.33788 | 0.40811 | 0.01917 |
|  | 2-Phenylpropionate | 0.03050 | 0.02084 | 0.18557 | 0.22506 | 0.00505 |
|  | 3-Chlorotyrosine | 0.12084 | 0.07606 | 0.68044 | 0.82332 | 0.00691 |
|  | 3-Hydromuconate | 0.05321 | 0.03366 | 0.30897 | 0.37321 | 0.02701 |
|  | 3-Hydroxybutyrate | 0.06711 | 0.03599 | 0.32755 | 0.39408 | 0.00039 |
|  | 3-Hydroxymandelate | 0.08662 | 0.05428 | 0.53336 | 0.64118 | 0.03491 |
|  | 3-Hydroxyphenylacetate | 0.02494 | 0.01803 | 0.15852 | 0.19249 | 0.00201 |
|  | 3-Indoxylsulfate | 0.02322 | 0.01922 | 0.16850 | 0.20537 | 0.00383 |
|  | 3-Methylxanthine | 0.01641 | 0.01181 | 0.10525 | 0.12795 | 0.00413 |
|  | 3-Phenyllactate | 0.06669 | 0.04407 | 0.38818 | 0.46986 | 0.02480 |
|  | 4-Aminohippurate | 0.04356 | 0.03104 | 0.27233 | 0.33059 | 0.00317 |
|  | 4-Hydroxybenzoate | 0.02161 | 0.01750 | 0.15344 | 0.18683 | 0.00387 |
|  | 4-Hydroxyphenylacetate | 0.03536 | 0.02464 | 0.21768 | 0.26416 | 0.00204 |
|  | 4-Hydroxyphenyllactate | 0.06252 | 0.03923 | 0.34675 | 0.41930 | 0.02771 |
|  | 5,6-Dihydrouracil | 0.01839 | 0.00969 | 0.08893 | 0.10698 | 0.00349 |
|  | 5-Hydroxyindole-3-acetate | 0.02263 | 0.01577 | 0.13932 | 0.16908 | 0.00205 |
|  | 5-Methoxysalicylate | 0.01883 | 0.01335 | 0.11717 | 0.14218 | 0.00204 |
|  | Acetamide | 0.04020 | 0.03022 | 0.26704 | 0.32453 | 0.00385 |
|  | Acetaminophen | 0.02438 | 0.01849 | 0.16291 | 0.19803 | 0.00404 |
|  | Acetoacetate | 0.01776 | 0.00934 | 0.08638 | 0.10397 | 0.01721 |
|  | Acetylsalicylate | 0.02312 | 0.01724 | 0.15362 | 0.18664 | 0.00857 |
|  | Adenine | 0.00823 | 0.00760 | 0.06711 | 0.08208 | 0.00496 |
|  | Asparagine | 0.16628 | 0.10283 | 0.91746 | 1.10872 | 0.04837 |
|  | Benzoate | 0.01603 | 0.01358 | 0.11925 | 0.14544 | 0.00468 |
|  | Carnosine | 0.29993 | 0.18822 | 1.66359 | 2.01333 | 0.03162 |
|  | Cinnamate | 0.03779 | 0.03064 | 0.26808 | 0.32647 | 0.00351 |
|  | Creatine | 0.01576 | 0.00873 | 0.07850 | 0.09457 | 0.03263 |
|  | Cystathionine | 0.14007 | 0.07500 | 0.68481 | 0.82389 | 0.00031 |
|  | Cystine | 0.12701 | 0.07098 | 0.63270 | 0.76238 | 0.04344 |
|  | Cytosine | 0.03260 | 0.02382 | 0.21015 | 0.25524 | 0.03060 |
|  | Ethanolamine | 0.04443 | 0.02454 | 0.22075 | 0.26586 | 0.03198 |
|  | Ferulate | 0.03744 | 0.02711 | 0.23740 | 0.28827 | 0.00240 |
|  | Gentisate | 0.01510 | 0.01189 | 0.10380 | 0.12628 | 0.00294 |
|  | Glutamine | 0.11162 | 0.06901 | 0.62618 | 0.75703 | 0.02627 |
|  | Hippurate | 0.04463 | 0.03263 | 0.28681 | 0.34852 | 0.00515 |
|  | Histamine | 0.09153 | 0.06573 | 0.59020 | 0.71726 | 0.00733 |
|  | Histidine | 0.13888 | 0.08737 | 0.78087 | 0.94447 | 0.02010 |
|  | Homocystine | 0.08435 | 0.04432 | 0.40617 | 0.48856 | 0.00233 |
|  | Homogentisate | 0.01681 | 0.01190 | 0.10462 | 0.12692 | 0.00244 |
|  | Imidazole | 0.06095 | 0.05217 | 0.45876 | 0.55979 | 0.00447 |
|  | Indole-3-acetate | 0.03769 | 0.02913 | 0.25813 | 0.31450 | 0.00305 |
|  | Isocitrate | 0.11844 | 0.06442 | 0.58387 | 0.70301 | 0.00274 |
|  | Isoeugenol | 0.03664 | 0.02519 | 0.22255 | 0.26986 | 0.03305 |
|  | Kynurenate | 0.01554 | 0.01335 | 0.11741 | 0.14329 | 0.00484 |
|  | Malate | 0.06560 | 0.03616 | 0.32213 | 0.38783 | 0.00165 |
|  | Mandelate | 0.05326 | 0.03527 | 0.33115 | 0.39969 | 0.01449 |
|  | N-Acetylglycine | 0.02135 | 0.01384 | 0.12401 | 0.15034 | 0.00550 |
|  | Niacinamide | 0.04102 | 0.03594 | 0.31651 | 0.38650 | 0.00466 |
|  | Nicotinate | 0.01443 | 0.01316 | 0.11651 | 0.14249 | 0.00465 |
|  | Pantothenate | 0.11032 | 0.06030 | 0.55270 | 0.66600 | 0.00698 |
|  | Phenol | 0.02286 | 0.01813 | 0.15860 | 0.19301 | 0.00327 |
|  | Phenylacetate | 0.03049 | 0.02216 | 0.19572 | 0.23785 | 0.00236 |
|  | Phenylacetylglycine | 0.06703 | 0.04641 | 0.40936 | 0.49676 | 0.00182 |
|  | Phenylalanine | 0.06490 | 0.04064 | 0.36201 | 0.43772 | 0.01378 |
|  | Phthalate | 0.01568 | 0.01303 | 0.11418 | 0.13915 | 0.00402 |
|  | Proline | 0.08064 | 0.04171 | 0.38938 | 0.46846 | 0.02644 |
|  | Protocatechuate | 0.01231 | 0.00985 | 0.08615 | 0.10486 | 0.00328 |
|  | Quinolinate | 0.00961 | 0.00836 | 0.07361 | 0.08986 | 0.00471 |
|  | Salicylate | 0.01336 | 0.01080 | 0.09454 | 0.11511 | 0.00386 |
|  | Salicylurate | 0.06829 | 0.04994 | 0.43747 | 0.53136 | 0.00309 |
|  | Sarcosine | 0.01545 | 0.00829 | 0.07558 | 0.09098 | 0.00734 |
|  | Taurine | 0.11453 | 0.06210 | 0.56442 | 0.67988 | 0.03159 |
|  | Theophylline | 0.01975 | 0.01292 | 0.11583 | 0.14042 | 0.00437 |
|  | Thymol | 0.03337 | 0.02034 | 0.18533 | 0.22387 | 0.04713 |
|  | Tiglylglycine | 0.03535 | 0.02288 | 0.20694 | 0.25006 | 0.04934 |
|  | Trigonelline | 0.01755 | 0.01308 | 0.11323 | 0.13790 | 0.00534 |
|  | Trimethylamine N-oxide | 0.02009 | 0.01068 | 0.09770 | 0.11751 | 0.01840 |
|  | Tyramine | 0.04592 | 0.03151 | 0.28215 | 0.34169 | 0.02733 |
|  | Tyrosine | 0.09036 | 0.05438 | 0.48765 | 0.58897 | 0.03777 |
|  | Urocanate | 0.04990 | 0.04077 | 0.35775 | 0.43587 | 0.00368 |
|  | Vanillate | 0.02488 | 0.01718 | 0.15073 | 0.18282 | 0.00333 |
|  | Xanthine | 0.00957 | 0.00840 | 0.07399 | 0.09033 | 0.00501 |
|  | o-Cresol | 0.01587 | 0.01168 | 0.10355 | 0.12581 | 0.00345 |
|  | p-Cresol | 0.02150 | 0.01592 | 0.14093 | 0.17118 | 0.00431 |
|  | Pi-Methylhistidine | 0.22613 | 0.14168 | 1.26088 | 1.52524 | 0.00831 |
|  | Tao-Methylhistidine | 0.16909 | 0.10401 | 0.93077 | 1.12511 | 0.01421 |


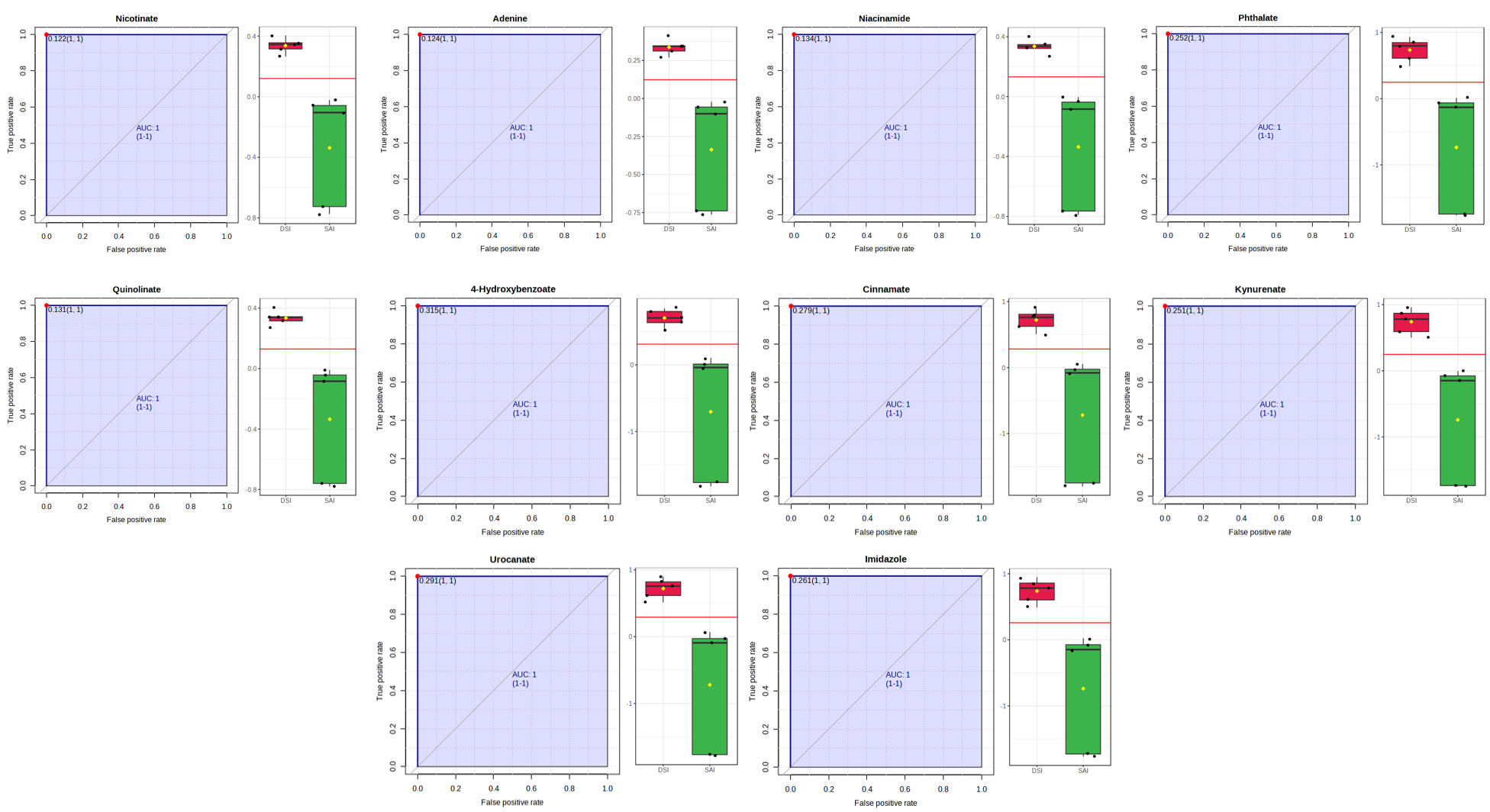
**Supplementary Figure 1.** The potential biomarkers in epidermis at 24 hpi. AUC, Area under curve; SAI, Saline-injection group; DSI, CHH dsRNA injection group.


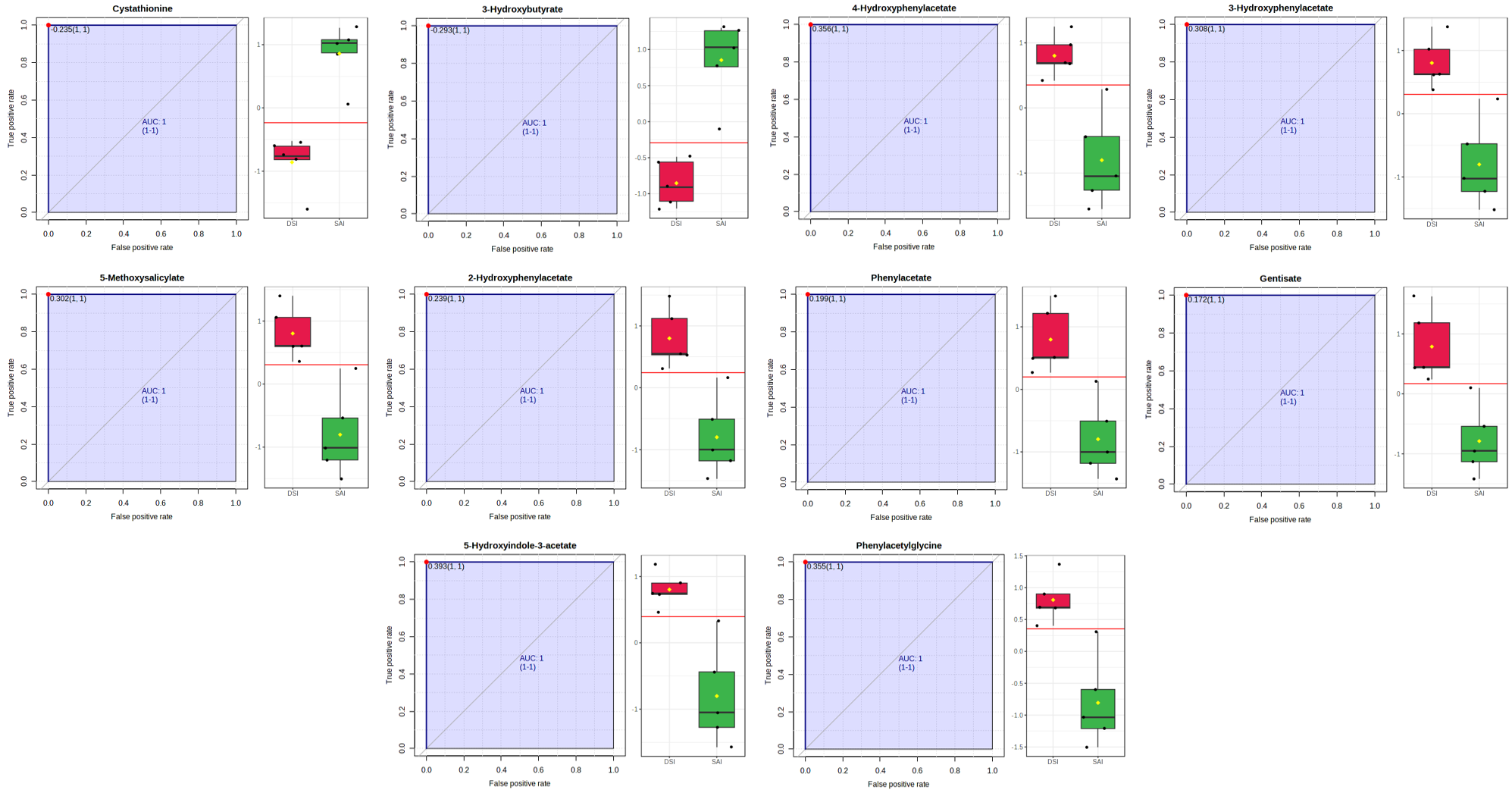
**Supplementary Figure 2.** The potential biomarkers in gills at 48 hpi. AUC, Area under curve; SAI, Saline-injection group; DSI, CHH dsRNA injection group.
